# Mutation of a single residue, β-glutamate-20, alters protein–lipid interactions of light harvesting complex II

**DOI:** 10.1111/j.1365-2958.2007.06017.x

**Published:** 2007-11-22

**Authors:** Lee Gyan Kwa, Dominik Wegmann, Britta Brügger, Felix T Wieland, Gerhard Wanner, Paula Braun

**Affiliations:** 1 Department Biologie I der LM-Universität München Botanik, 80638 München, Germany.; 2 Biochemie-Zentrum der Universität Heidelberg Im Neuenheimer Feld 328, 69120 Heidelberg, Germany.

## Abstract

It is well established that assembly of the peripheral antenna complex, LH2, is required for proper photosynthetic membrane biogenesis in the purple bacterium *Rhodobacter sphaeroides*. The underlying interactions are, as yet, not understood. Here we examined the relationship between the morphology of the photosynthetic membrane and the lipid–protein interactions at the LH2–lipid interface. The non-bilayer lipid, phosphatidylethanolamine, is shown to be highly enriched in the boundary lipid phase of LH2. Sequence alignments indicate a putative lipid binding site, which includes β-glutamate-20 and the adjacent carotenoid end group. Replacement of β-glutamate-20 with alanine results in significant reduction of phosphatidylethanolamine and concomitant raise in phosphatidylcholine in the boundary lipid phase of LH2 without altering the lipid composition of the bulk phase. The morphology of the LH2 housing membrane is, however, unaffected by the amino acid replacement. In contrast, simultaneous modification of glutamate-20 and exchange of the carotenoid sphaeroidenone with neurosporene results in significant enlargement of the vesicular membrane invaginations. These findings suggest that the LH2 complex, specifically β-glutamate-20 and the carotenoids' polar head group, contribute to the shaping of the photosynthetic membrane by specific interactions with surrounding lipid molecules.

## Introduction

In recent years, the understanding of membrane proteins' structure and function has tremendously been advanced. The interplay of lipids and membrane proteins has been recognized to be vital for maintaining and optimizing their functions (for recent reviews see, e.g. Dowhan *et al*., 2004; Jones, 2007). The ways through which membranes are formed into particular shapes has gained recent attention. The number of factors known to be involved in membrane budding and maintenance of particular membrane shapes is continually raising and has given some insight into the complexity of these processes.

The role of lipid-specific dynamics in enabling or generating membrane curvature has been an area of challenging research (for recent reviews see, e.g. Farsad and De Camilli, 2003; Antonny *et al*., 2005). Certain lipid species are postulated to favour bilayer curvature owing to their physicochemical properties, their relative geometries, or both (e.g. Gruner, 1985; Chernomordik and Zimmerberg, 1995). Such non-bilayer lipids induce, owing to their conical shape, local non-planar structures in bilayered lipid membranes. Non-bilayer lipids include phosphatidylethanolamine (PE), a major phospholipid in many organisms, and monogalactosyldiacylglycerol, a major phospholipid in the inner chloroplast membrane. Functional membranes such as photosynthetic or mitochondrial membranes contain high amounts of non-bilayer lipids. The concentration of these lipids is precisely regulated which points to their critical function (Wieslander *et al*., 1980). Currently, several functions are being discussed. Among those are a role in keeping integral membrane proteins in a functional state (for a recent review see, De Kruijff, 1997), maintenance of particular membrane properties by regulation of the protein lipid ratio (for a recent review see, Garab *et al*., 2000) or mediation of dynamic membrane properties such as membrane fusion events or stacking/unstacking of thylakoid membranes (see, e.g. Simidjiev *et al*., 1998; Lee, 2000).

Selective transfer of non-bilayer lipids between bilayer leaflets has been proposed as a means by which surface area asymmetries could influence membrane curvature and budding (for recent reviews see, e.g. Siegenthaler, 1998). Another effect of such lipids is loose packing of the lipid head groups which promotes partitioning of polypeptide into the membrane's interface and thus the interactions essential for, e.g. protein import (for recent reviews see, e.g. De Kruijff *et al*., 1998; van Dalen and De Kruijff, 2004) or membrane deforming (Farsad and De Camilli, 2003; Antonny *et al*., 2005).

The contribution of proteins to the shaping of membranes is by contrast just beginning to be unravelled (for recent reviews see Farsad and De Camilli, 2003; Antonny *et al*., 2005; Shibata *et al*., 2006). Membrane associated proteins can induce membrane curvature by asymmetric penetration of the bilayer and alterion of the relative bilayer surface areas. Others appear to selectively bind lipids and thereby contribute to their enrichment into one leaflet of the membrane. Many issues are not clear, but it is likely that the process is driven by a cooperation of both proteins and lipids.

Considerable knowledge has been accumulated on this in the plant field. The major chloroplast proteins have been shown to force the non-bilayer lipids to adopt a bilayer structure, and the combination of these proteins and the lipids drives the formation of the thylakoid membrane stacks (for a recent review see e.g. Lee, 2000). Self-assembly of ordered lamellar membrane structures *in vitro* may be induced by association of protein and lipid components of the thylakoid membrane (Simidjiev *et al*., 2000). The molecular interactions underlying these processes, however, are largely not yet known.

Three types of binding modes have been defined for lipid interactions with membrane proteins (see, e.g. Palsdottir and Hunte, 2004; Hunte, 2005). The ‘integral protein lipids’ reside usually within a membrane protein or a membrane protein complex. A shell of ‘annular lipids’ bound to the protein surface resembles the bilayer structure. ‘Non-annular surface lipids’ are immersed in cavities and clefts of the protein surface. They are frequently observed for multisubunit complexes and multimeric assemblies and are typically present at contact sites between adjacent subunits. Because of the high level of functional and structural information available for photoactive membrane proteins they frequently serve as models for lipid–protein interactions (for a recent review see, e.g. Pali *et al*., 2003).

Photosynthetic bacteria exhibit a large variety of photosynthetic membrane morphologies (Wanner *et al*., 1986; Drews and Niederman, 2002). The purple non-sulphur bacteria produce a specialized intracytoplasmic membrane (ICM) comprised of interconnected buds which harbour light-harvesting (LH) complexes, usually the peripheral antenna, LH2, LH1 and reaction centres (RC) (reviewed in Drews and Oelze, 1981; Kiley and Kaplan, 1988) in unique macromolecular arrangements (Jungas *et al*., 1999; Frese *et al*., 2000; Scheuring *et al*., 2004). ICM formation is repressed by high oxygen tension under chemoheterotrophic conditions, while lowering the oxygen partial pressure in the dark results in ICM biogenesis by invagination of the cytoplasmic membrane, together with the synthesis and assembly of LH and RC. The ICM development is under the control of a global two-component oxygen sensing, signal transduction system (Sganga and Bauer, 1992; Phillips-Jones and Hunter, 1994; Eraso and Kaplan, 2000). The synthesis of bacteriochlorophyll a (BChl a), carotenoid (Car) and LH2 (Ponnampalam *et al*., 1995) is under the control of additional components including overlapping aerobic repressor circuits. A photoreceptor integrates both redox and light signals (Braatsch *et al*., 2002; Koblizek *et al*., 2005).

In *Rhodobacter sphaeroides*, which is principally used as model organism for photosynthetic purple bacteria, properly assembled peripheral LH2 complexes are required for the complete maturation of the ICM (Hunter *et al*., 1988; Sturgis and Niederman, 1996), whereas it is independent of the presence of RC or LH1 complexes (Hunter and Turner, 1988; Kiley *et al*., 1988). The LH2 are oligomeric complexes of elementary subunits comprised of two small single membrane-spanning polypeptides, the α- and β-subunits, which bind one Car and three BChl a molecules (McDermott *et al*., 1995; Koepke *et al*., 1996; Papiz *et al*., 2003). The nine α-apoproteins form an inner hollow cylinder, the nine β-apoproteins an outer cylinder and most of the photoactive pigments are sandwiched in between. The entire LH2 complex is embedded in the membrane as a cylindrical structure of ∼7 nm in diameter and ∼4 nm in height (McDermott *et al*., 1995), and extends by 1.0 nm from the lipid bilayer on the cytoplasmic side and 0.2 nm from the periplasmic side (Stamouli *et al*., 2003). It has been speculated that LH2 complex specifically interacts with phospholipids which contribute to the ICM formation in an, as yet, poorly understood manner (Russell *et al*., 2002). Hitherto, neither structural nor annular lipids have been detected in the crystal structures of the LH2 complexes (McDermott *et al*., 1995; Koepke *et al*., 1996; Papiz *et al*., 2003).

Carotenoids have been shown to be essential for the purple bacterial membrane morphogenesis. Mutants with disruptions in the Car biosynthesis have shown gross alterations in ICM morphology (Lommen and Takemoto, 1978; Lang and Hunter, 1994). The assembly of stable LH2 appears to require the presence of Cars as purple, non-sulphur bacteria that are Car-deficient generally also lack LH2 complexes (Cohen-Bazire and Stanier, 1958; Fuller and Anderson, 1958; Hunter *et al*., 1994; Lang and Hunter, 1994). The Car-less *R. sphaeroides* mutant, R26 (Clayton and Smith, 1960), entirely lacks LH2. This mutant, however, has a tendency to revert to the strain R26.1, containing a profoundly modified LH2 complex which still lacks Cars. The additional modification somewhat compensates for the absence of Car. It is still not clear whether Cars directly assert their effects on membrane biogenesis or indirectly owing to their role in LH2 stability.

Early investigations of membrane phospholipid composition of *R. sphaeroides* 2.4.1 (LH2^+^ lH1^+^ RC^+^) grown photosynthetically (e.g. Takemoto and Lascelles, 1973; Birrell *et al*., 1978; Russell and Harwood, 1979; Albayatti and Takemoto, 1981; Onishi and Niederman, 1982) had suggested that the major phospholipids are PE, phosphatidylglycerol (PG) and phosphatidylcholine (PC), and the minor lipids are cardiolipin and phosphatidic acid. The relative proportions of these phospholipids quoted in previous works, however, differed significantly. Although it is well established that the proper development of the vesicular ICM depends on the LH2 complex, the contribution of the lipids is not understood. The molecular features driving membrane budding and shaping are, yet, still unknown. To start unravelling the mechanism of membrane shaping by the antenna complex, we studied LH2–lipid interactions. We found that PE is specifically accumulated largely at the LH2–lipid interface, and that the PE accumulation depends on a single residue, β-glutamate-20. We showed that alteration (β-Glu-20) of this LH2–lipid interface, in particular, β-glutamate-20 and the adjacent Car modified the shape of the membrane invaginations. The results are discussed in relation to binding of boundary lipids and lipid membrane shaping.

## Results and discussion

### Mutagenesis of LH2 affects ICM membrane morphology

**Fig. 1:**
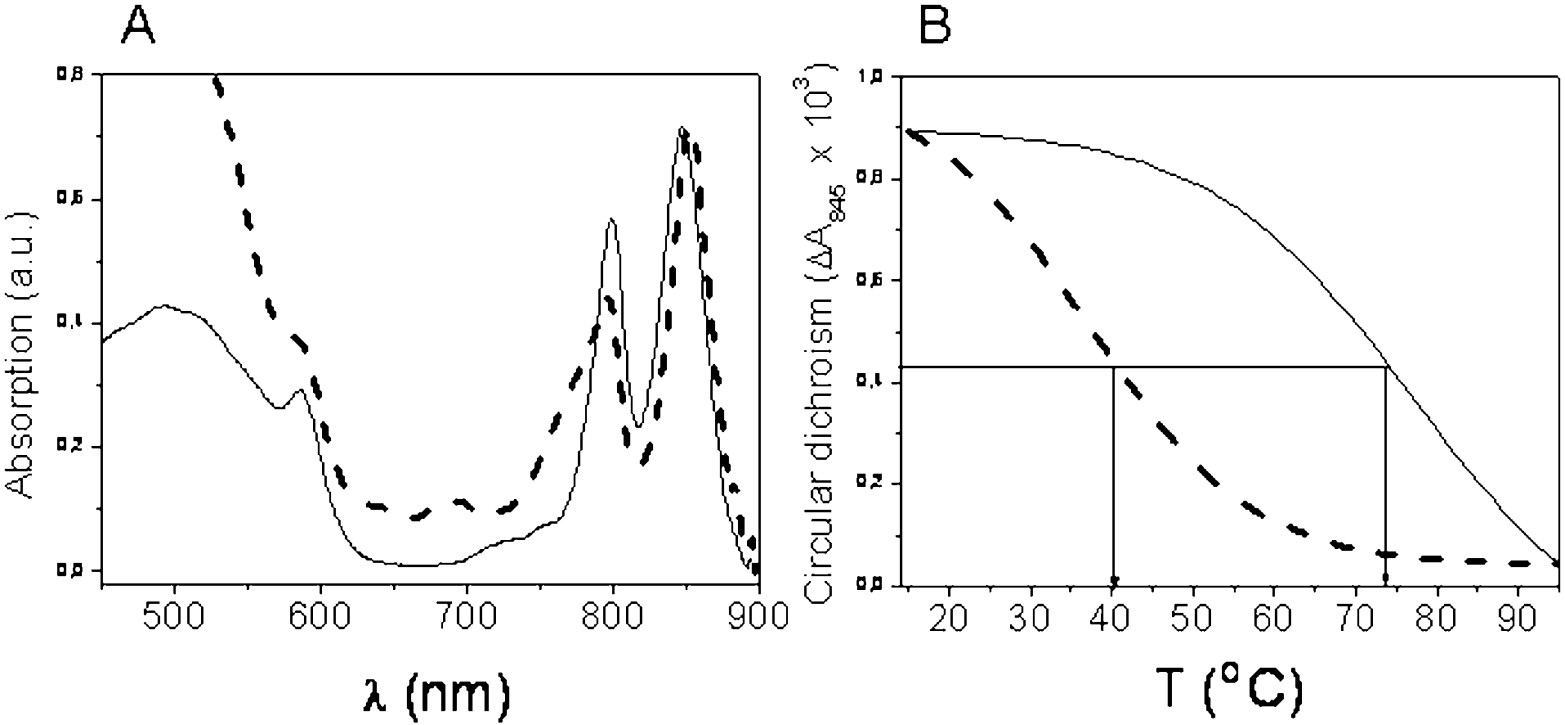
Assembly of LH2 wt and massively mutated LH2 αAL_16-4S_/βAL_12_ of *R. sphaeroides*. A. Absorption spectra of LH2 WT (–) and αAL_16-4S_/βAL_12_ (---). Spectra are normalized at 850 nm. B. Thermal denaturation of LH2 WT (–) and LH2 αAL_16-4S_/βAL_12_ (---). Changes of the CD signal at 845 nm during heating of suspended LH2 membranes are monitored. The T_m_ values are indicated by the arrow.

To examine the morphogenesis of the ICM membrane in dependence of LH2 assembly, LH2 wild type (wt) or mutant complexes are expressed in a deletion strain of *R. sphaeroides* (LH2^-^ LH1^-^ RC^-^). This strain is devoid of endogenous BChl-binding proteins 1 but capable of BChl synthesis (Jones *et al*., 1992). Changes in ICM development may thus be directly correlated with changes in expression and/or assembly of LH2 owing to the absence of additional BChl-binding complexes (LH1 and RC). The first mutant of which we studied the membrane topology is LH2 αAL_16-4S_/βAL_12_ (Table 1). We have previously shown that the stable assembly of the LH2-like complex is severely affected by massive mutagenesis of the chromphor binding site (Kwa *et al*., 2004). The LH2 absorption spectra with red most absorption bands of the BChl-B850^2^ and BChl-B800 are at ∼849 and 800 nm (Fig. 1A) and thus typical for the spectra of the antenna complex from *R. sphaeroides* (see, e.g. Cogdell and Scheer, 1985; van Grondelle, 1985; Braun and Scherz, 1991). The structural stability of LH2 αAL_16-4S_/βAL_12_, however, is significantly impaired as compared with the one of LH2 wt (Fig. 1B). In addition, the LH2 complex concentration relative to total protein concentration in membranes of LH2 αAL_16-4S_/βAL_12_ is approximately six times lower than that of membranes containing LH2 wt (Table 2).

**Table 2:** Comparison of LH2 wt and mutant expression levels.

	Total membrane protein content (μg/ml)		Expression level (LH2 mutant/LH2 Wt)	
		
	DD13	DG2	DD13	DG2
LH2 WT	117	125		
LH2 αAL_16-4S_/βAL_12_	680	n.d.	5,8	n.d.
LH2 αWT/βWT_−20A_	150	190	1,3	1,5

Total protein content is given of membranes adjusted to OD_850_ = 1. Relative expression levels are obtained by ratios of total protein in LH2 mutant to total protein in LH2 wt.

n.d., not determined.

**Table 1:** Aa sequences of TM stretches of αβ-polypeptides of LH2 wt and mutants used in this study.

LH2	α-subunit	β-subunit
WT	TVGVPLFLSAAVIASVVIHAAVLTTT	AEEVHKQLILGTRVFGGMALIAHFLAAAA
αAL_16-4S_/βAL_12_	TVGVPLFLSAA**LL**AS**LLI**HAA**L**L**AA**T	AEEVHKQLILGTRVF**LLI**AL**L**AHLLAAAA
αWT/βWT_−20Q_	TVGVPLFLSAAVIASVVIHAAVLTTT	AE**Q**VHKQLILGTRVFGGMALIAHFLAAAA
αWT/βWT_−20A_	TVGVPLFLSAAVIASVVIHAAVLTTT	AE**A**VHKQLILGTRVFGGMALIAHFLAAAA
αWT/βWT_−20K_	TVGVPLFLSAAVIASVVIHAAVLTTT	AE**K**VHKQLILGTRVFGGMALIAHFLAAAA

Aa replacements are shown in bold. The histidine ligand, designated His 0 of the central magnesium of the BChl-850 is underlined.

**Fig. 2:**
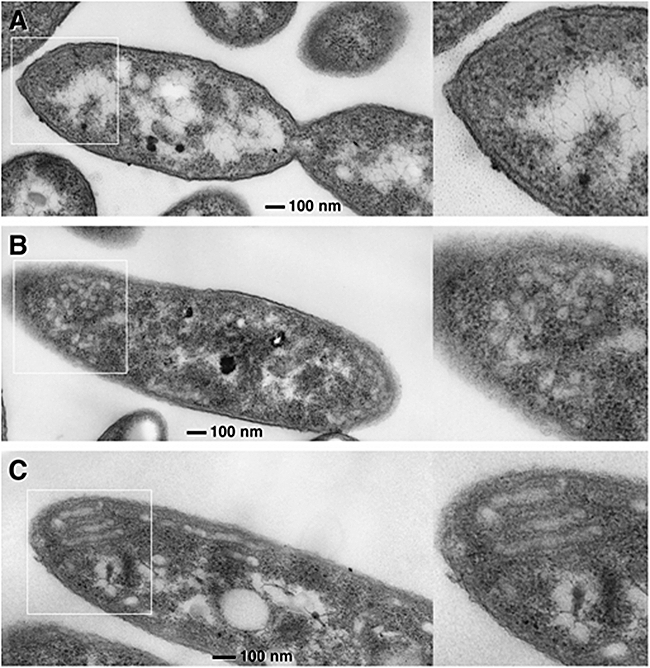
Transmission electron micrograph of ultrathin sections of *R. sphaeroides* DD13 cells lacking LH2 (A) expressing LH2 WT (B) or LH2 αAL_16-4S_/βAL_12_ (C) without invaginations, with ‘normal’ vesicular invaginations and ‘abnormal’ tubules respectively. On the right, the enlarged view of the respective structures.

The ultrastructures of ICM of *R. sphaeroides* DD13 cells not expressing LH2, expressing LH2 wt and model LH2 αAL_16-4S_/βAL_12_, are shown in Fig. 2. In the absence of LH2 complexes, vesicular ICM invaginations are not observed, while in the presence of LH2 wt complexes mature ICM is observed with vesicle-like invaginations ranging from 35 to 55 nm in diameter. The size and shape of the vesicular invaginations are similar to those reported previously for *R. sphaeroides* 2.4.1 cells grown photosynthetically and containing LH2, LH1 and RC (Gibson, 1965; Fraker and Kaplan, 1972; Kiley and Kaplan, 1988; Feniouk *et al*., 2002). In contrast, the radii of the ICM invaginations containing αAL_16-4S_/βAL_12_ are significantly enlarged relative to the invaginations of ICM containing LH2 wt, and moreover they are changed from normal spherical to tubular shaped membranes of up to a few hundred nanometres in length (Fig. 2C). Apparently, LH2 properties and expression level are correlated with the size and morphology of ICM in deletion strain DD13 devoid of core complexes. Previously, it has been shown that the absence of LH2 but presence of LH1 and RC results in tubular ICM in *R. sphaeroides*. Here, it is shown that tubular ICM, albeit of smaller size, also develop in the presence of massively mutated LH2 complexes.

### Alteration in membrane morphology correlates with altered phospholipid composition

To determine whether the altered membrane morphology is accompanied by changes in the membrane lipids, the phospholipid compositions of ICM containing LH2 wt or αAL_16-4S_/βAL_12_ complex were determined by ESI-MS/MS as described in Brugger *et al*. (1997; 2006). The phospholipid composition of cellular membrane, i.e. of intact cells, and of isolated chromatophores did generally not significantly differ (data not shown), which is similar to previous findings obtained from studies with *R. sphaeroides* (Onishi and Niederman, 1982). Largely, in line with previous studies (Russell and Harwood, 1979; Onishi and Niederman, 1982) PE, PC and PG make up the major phospholipids in *R. sphaeroides* (Table 3), while others are present only in trace amounts. PE is the most abundant phospholipid in the cellular membranes of *R. sphaeroides* strain DD13 (Table 3). In *R. sphaeroides* DD13 expressing LH2 wt, PE is slightly increased as compared with cells not expressing LH2 (∼61 ± 2.2% versus ∼58 ± 0.8%) whereas PC is reduced (from 27 ± 0.7% to ∼23 ± 0.6%). The relative amount of PG makes up less than 20% in all cases and thus does not change remarkably in mutant or wt membranes. This differs from previous studies, which reported on a significant increase in PG upon photosynthetic growth in *R. sphaeroides* 2.4.1 (Russell and Harwood, 1979). In a very recent MS analysis of the cell lipids from *Rhodopseudomonas acidophila*, PG has been shown to even decrease upon induction of photosynthetic growth to a value as low as 11–16% (Russell *et al*., 2002). Remarkably, the phospholipid composition of cells expressing αAL_16-4S_/βAL_12_ is dramatically changed. The relative PE content has dropped by ∼13.5% while the PC and PG content rose by ∼25.6% and ∼13.8% respectively. The PC+PG/PE ratio is thus increased from 0.65 in *R. sphaeroides* strain DD13 expressing LH2 wt to 0.90 in cells expressing αAL_16-4S_/βAL_12_. Thus, mutagenesis of the LH2 proteins not only results in a significant reduction in the complexes' expression level and a change in membrane morphology but also in a change of the overall phospholipid composition, in particular, the bilayer to non-bilayer lipid ratio.

**Table 3:** Relative content of major phospholipids in *R. sphaeroides* DD13 cells lacking LH2 (DD13) expressing LH2 WT or LH2 αAL_16-4S_/βAL_12_.

	Percentage of phospholipid			
		
	PC	PE	PG	PC+ PG/PE
DD13^a^	27.2 ± 0.7	58.2 ± 0.8	14.6 ± 1.5	0.72
LH2 WT^b^	23.4 ± 0.6	60.7 ± 2.2	15.9 ± 2.8	0.65
αAL_16-4S_/βAL_12_^b^	29.4 ± 2.6	52.5 ± 5.6	18.2 ± 3	0.90

a Average values derived from at least two measurements from two independent samples.

b Average values derived from at least four measurements from three independent samples.

The relative content of PC, PE and PG are determined by ESI-MS/MS and expressed as percentage of their combined total.

**Fig. 3:**
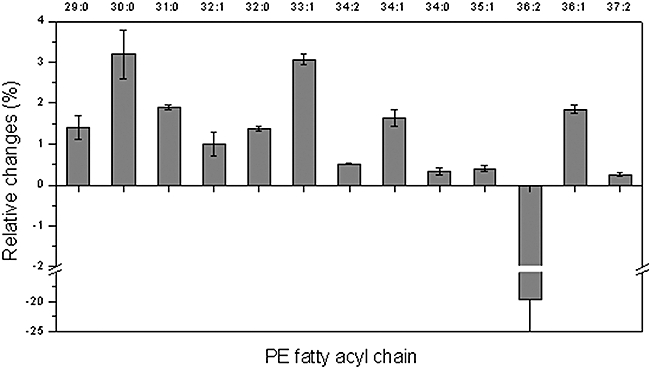
Changes in PE acyl chain composition in cellular membranes of *R. sphaeroides* DD13 expressing LH2 WT and LH2 αAL_16-4S_/βAL_12_. The difference in acyl chain content is shown. The average values are derived from at least six measurements of three independent samples. Note, the significant decrease (∼20%) in 36:2 tail content in LH2 αAL_16-4S_/βAL_12_.

Both the ratio of bilayer/non-bilayer lipids and fatty acyl chain length have been implied to partake in regulation of membrane curvature energy (e.g. Vikstrom *et al*., 2000). The fatty acyls of *R. sphaeroides* DD13 devoid of LH2, expressing LH2 wt or αAL_16-4S_/βAL_12_, were not analysed in detail but only characterized by the total number of carbons (ΣC) and double bonds (Σ). In all three samples, the major fatty acyl chain composition is 36:2, which is likely to be composed of two fatty acids and one double bond each (C18:1). Vaccenic acid, 18:1Δ11, makes up > 80% of the lipid tails in photosynthetically grown *R. sphaeroides* 2.4.1 and *R. capsulatus* (Russell and Harwood, 1979), and 45% in *Rps. acidophila* (Russell *et al*., 2002). The acyl-tails of the lipids of *R. sphaeroides* DD13 show only minor variations upon ICM development; neither the amount nor the degree of saturation varied significantly (data not shown). Similarly, the acyl-chains of PC which consist primarily of C18:2 are not altered upon expression of LH2. On the other hand, the composition of the PE acyl chains is significantly altered in cells expressing αAL_16-4S_/βAL_12_ as compared with cells expressing LH2 wt (Fig. 3). The major PE acyl-chain 36:2 is reduced from ∼79% in DD13 cells expressing LH2 wt to ∼61% in cells expressing αAL_16-4S_/βAL_12_. Concurrently, saturated lipids with less carbons (ΣC:ΣΔ = 29:0, 30:0, 31:0, 32:1, 32:0 and 33:1) are increased (Fig. 3). These shorter acyl-chains were either untraceable and/or present in only scarce amounts in cells expressing LH2 wt. The hydrocarbon composition of PC shows only minor variations (not shown), indicating that exclusively the acyl composition of PE is modulated upon alterations of the LH2 expression level and ICM morphology in *R. sphaeroides* DD13.

Taken together, our findings indicate that interactions between the lipids and LH2 influence the properties of the ICM. Pronounced changes in morphology of the ICM containing modified LH2 are accompanied by changes in the phospolipid composition. The relative content of bilayer lipids is increased as well as the content of shorter, fully saturated fatty acyl chains of these lipids. Both changes concur with the more planar morphology of the ICM in cells expressing αAL_16-4S_/βAL_12_. Membranes of cells not expressing LH2 and cells expressing LH2 wt, however, have a similar hydrocarbon tail composition, indicating that the lipid tail length and saturation do not play a critical role in formation and maintenance of ICM in the presence of native LH2.

### Residue β-glutamate-20 contributes to PE accumulation at the LH2–lipid interface

**Fig. 4:**
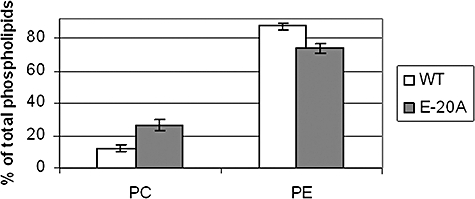
Phospholipid compositions of isolated LH2 WT and LH2 αWT/βWT_−20A_. For experimental details see Table 3.

Recently, evidence is accumulating that conserved lipid-binding motifs exist (Wakeham *et al*., 2001; Palsdottir *et al*., 2003; Palsdottir and Hunte, 2004). To identify aa residues of the LH2 polypeptides which potentially partake in specific LH2–lipid interactions, a number of different strategies have been pursued. In the TM stretches of the α- and β-polypeptides of bacterial light harvesting complexes, relatively few residues are found to be highly conserved (Zuber, 1986; Braun *et al*., 2002). In 34 different β-subunits only the residues at position 0, −4 and −8 are noted to be strictly conserved. Conspicuously, at the N-terminal domain of the β-subunit the glutamate residues at position −20 and −23 are also conserved in both LH2 and LH1 β-subunits even from such relatively remote genera as Erythrobacter and Chromatium. In the high-resolution structure of LH2 from *Rps. acidophila* (McDermott *et al*., 1995; Papiz *et al*., 2003), βGlu-20 is neither in close contacts with BChls pigments nor with residues from neighbouring subunits, and is thus not obviously involved in protein–protein or BChl–protein interactions. It is in close contact, however, with the Car, in particular, with atoms of its polar head groups. βGlu-20 is located at the cytoplasmatic end of the β-TMH, at the outer surface of the cylindrical structure of the LH2. It is therefore likely in contact with the membrane lipids, particularly, with the headgroups (Prince *et al*., 2003). Thus, βGlu-20 has been replaced by glutamine, alanine or lysine which did not affect the functional assembly of LH2 (see below), and the mutant complexes have been analysed for their interactions with the immediate lipid environment. To that end the LH2 wt and mutant lipid compositions of cellular membranes and of the boundary lipids, i.e. the lipids which remain closely attached to the complex upon purification from the membrane have been analysed (Table 4). Remarkably, the composition of LH2 boundary lipids is distinctively different from the composition of bulk lipids as present in the cellular membranes. PE is clearly crowding at the LH2–lipid interface, constituting as much as 88% of the total phospholipids (as compared with 61% in bulk phase). PC makes up only 12%, and PG is below detection level. It should be noted that the composition of the LH2 boundary lipids as determined here may depend on the experimental conditions, for example, the detergent used for extraction from the ICM. We have roughly estimated the molar ratios of lipids to LH2: in isolated complexes five to six lipid molecules are found per LH2 complex. Thus, of the nine potential PE sites, nearly about 60% appear occupied by a lipid molecule even after detergent treatment. Out of the lipids that are still attached ∼90% are PE molecules, thus less than one of the potential PE sites may have a PC attached (PC content is ∼10% of the 5–6 phospholipids found attached to isolated LH2). Upon replacement of Glu-20 with Ala, PE remains clearly the major phospholipid closely associated with LH2 αWT/βWT_−20A_; however, the relative amount of PE is reduced by ∼17% in comparison to wt LH2 (from 88 ± 2.1% to 74 ± 3.1%) (Fig. 4; Table 4). PC is increased from 12% to 26%. In the LH2 αWT/βWT_−20A_ mutant, the total amount of lipids found attached remains roughly constant but the PE content is reduced by ∼17%, i.e. only three to four of the lipid binding sites still bind a PE. The number of sites occupied by PC molecules has apparently increased to two out of the six to seven sites which are occupied by lipid molecules.

**Table 4:** Cellular and boundary phospholipid compositions of LH2 WT and LH2 αWT/βWT_−20A_.

	Percentage of phospholipid		
	
	PC	PE	PG
LH2 WT cells^a^	23.4 ± 0.6	60.7 ± 2.2	15.9 ± 2.8
LH2 αWT/βWT_−20A_ cells^b^	23.9 ± 3.4	61.9 ± 4.6	14.16 ± 4.8
Isolated LH2 WT^a^	12.3 ± 2.1	87.7 ± 2.1	n.d.
Isolated LH2 αWT/βWT_−20A_^c^	26.2 ± 3.3	73.9 ± 3.1	n.d.

a Average values are derived from at least four measurements from three samples.

b Average values are derived from ten measurements from five samples.

c Average values are derived from two measurements from two independent samples.

n.d., not determined.

**Fig. 5:**
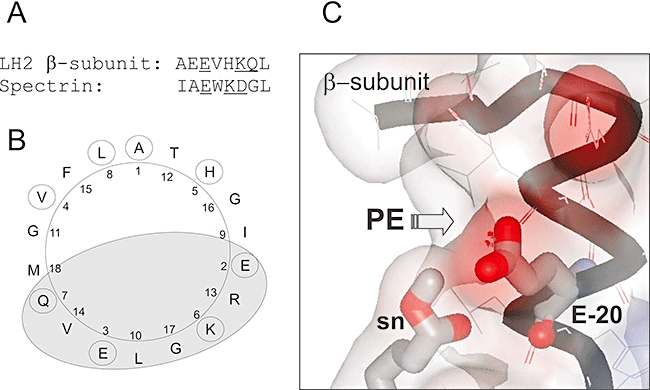
Putative PE binding site in LH2 complex from *R. sphaeroides*. A. Aa sequences of putative PE binding motifs in spectrin (Hryniewicz-Jankowska *et al*., 2004) and the LH2 β-subunit. Residues presumed to be involved in PE binding are underlined. B. Helical wheel representation of the TMH of the LH2 β-subunit. The residues within the sequence motif are circled. The eclipse indicates the presumed lipid binding surface. C. In the high-resolution structure (PDB file:1KZU) of *Rps. acidophila*, the rhodopin glucoside head group has been replaced with the sphaeroidenone head group. Energy minimization has been carried out on the replaced groups using the molecular modelling and visualization software WebLab ViewerPro 3.7 (Molecular Simulations Inc.). For clarity, only the β-TMH, sphaeroidenone and β-glutamate-20 are depicted in detail. Colouring is according to elements and in case of surface to electrostatic charge. The arrow points at the putative PE binding site.

In contrast to the boundary lipid composition, the bulk lipid composition of membranes containing αWT/βWT_−20A_ is not significantly affected by the substitution of glutamate with alanine (Table 4). The hydrocarbon chains of the PE boundary lipids with 36:2 acyl chains have conspicuously increased in the mutant by ∼20% relative to wt (Table 5). The tail composition of PE in close vicinity of αWT/βWT_−20A_ is hence similar to the tail composition of PE in bulk phase. The lipids that are attached to isolated αWT/βWT_−20A_ have thus been found altered as compared with the lipids attached to LH2 wt. The specificity of lipid–LH2 interaction is diminished by the replacement of glutamate −20 with alanine. It can be estimated that while in LH2 wt nearly all of the potential lipid binding sites have a PE molecule attached, in αWT/βWT_−20A_ one-third of the sites have a PC molecule attached. This indicates that the conserved residue glutamate −20 contributes to the crowding of PE at the LH2 lipid interface. The fully protonated ammonium group of PE is suited to electrostatically interact with the negatively charged side-chain of glutamate −20 contrary to the N^+^ of PC which is shielded by the three methyl groups. In addition, the ammonium group of PE has the capability of forming hydrogen bonds with the carboxy group of Glu, a property not shared with PC. The presence of a combined positively charged and polar residue, namely Lys-17 and Gln-16, in the immediate vicinity of Glu-20 further supports the notion that this stretch of the β-TMH constitutes a lipid-binding motif. Positively charged residues are present in lipid-binding motifs primarily at the n-side of the membrane (Palsdottir and Hunte, 2004). In case of spectrin the sequence stretch, IAEWKDGL appears to be essential for binding to PE enriched membranes as shown by deletion mutation studies (Hryniewicz-Jankowska *et al*., 2004). The lipid-binding motif comprises two negatively charged, one aromatic and one positively charged residue (Fig. 5A). However, single residue substitutions within the proposed motif have not been carried out and the significance of the single residues is not known. Similarly, the aa sequence of the N-terminal edge of the LH2 β-TMH contains residues with negatively and positively charged side-chain. In addition, a lysine residue in combination with a polar residue is frequently found in lipid-binding motifs (Wakeham *et al*., 2001; Palsdottir *et al*., 2003; Palsdottir and Hunte, 2004). The putative PE binding site is illustrated in the X-ray structure of *Rps. acidophila* (Fig. 5). Lys-17 and Gln-16 as well as Glu-20 are clearly making up one interface of the β-TMH (Fig. 5B) and thus are likely to constitute a lipid-binding surface within the proposed binding motif. PE was previously suggested to be selectively accumulated on the cytoplasmic face of the ICM in *R. sphaeroides* (Marinetti and Cattieu, 1981), which further supports the notion that PE specifically binds to the putative lipid-binding motif at the cytoplasmic edge of the β-subunit. Furthermore, LH complexes from *R. capsulatus* have been shown to be stably inserted into the membrane *in vitro* only when associated with PE (Pucheu *et al*., 1999). These findings also suggest a close interaction between LH2 and PE molecules.

**Table 5:** PE fatty acyl composition of boundary lipids of LH2 WT and LH2 αWT/βWT_−20A_.

	Percentage (%)	
	
Fatty acyl chain (ΣC: ΣΔ)	LH2 WT^a^	LH2 αWT/βWT_−20A_^b^
32:0	1.89	–
34:2	1.83	0.66
34:1	7.79	4.73
34:0	3.41	–
35:1	1.34	0.58
**36:2**	**59.03**	**71.25**
36:1	12.69	16.00
37:2	3.72	2.15
38:1	1.33	–
Others	6.97	1.07

a Average values are derived from four measurements from three independent samples.

b Average values are derived from two measurements from two independent samples.

Values are percentage of total fatty acyl chains of isolated LH2 complexes. Note the significant decrease (≥ 20 %) in the major 36:2 fatty acyl chain (indicated in bold).

### Residue β-glutamate-20 and Car synergystically contribute to LH2 assembly

As apparent from the LH2 high-resolution structure from *Rps. acidophila* (McDermott *et al*., 1995), βGlu-20 interacts with the Car molecule, rhodopin glucoside, particularly the glucoside moiety (Fig. 5). In *R. sphaeroides*, the major Car is not rhodopin glucoside but spheroidenone. These carotenoids have similar polyene chains but differ in their headgroups; in rhodopin glucoside it is the glucose group, in sphaeroidenone a methoxy and a keto group (see Fig. S1). An inspection of the X-ray structure indicates that these functional groups may be close to the conserved Glu-20 in the LH2 from *R. sphaeroides* (Fig. 5). To examine the effect of the Car polar end groups on LH2–lipid interactions, we set out to replace the native sphaeroidenone with neurosporene. In the *R. sphaeroides* mutant strain, DG2, the last step of the sphaeroidene biosynthesis is disrupted (Hunter *et al*., 1994). This strain has as major Car the biosynthetic precursor neurosporene, which lacks the polar end groups of spheroidenone, the methoxy and keto groups (see Fig. S1). To study the effect of the Car on the membrane properties, LH2 wt and mutant LH2 αWT/βWT_−20Q_, αWT/βWT_−20A_ and αWT/βWT_−20K_ were expressed in *R. sphaeroides* strain DG2 with neurosporene as major Car. Wt and mutant LH2 complexes have closely similar spectral properties indicating that the functional assembly of the LH2 mutant complexes is retained also in the presence of neurosporene instead of sphaeroidenone (Fig. S1). Some minor modifications are observed in the absorption spectra in the blue region of αWT/βWT_−20A_ owing to increased light scattering (Kwa *et al*., 2004). In addition, the fluorescence excitation spectra of αWT/βWT_−20A_ are slightly altered in comparison to the spectra of wt LH2. The energy transfer from Cars to BChls is somewhat reduced, particularly in αWT/βWT_−20A_, and there are minor alterations in the shape of the excitation spectrum which may be due to the increased scattering. However, none of the differences indicates that the functional assembly of the mutant complexes is significantly impaired by the combined alterations, the replacement of glutamate −20 with alanine, and of sphaeroidenone with neurosporene. The structural stabilities of wt LH2 and αWT/βWT_−20Q_, αWT/βWT_−20A_ and αWT/βWT_−20K_ containing neurosporene as assessed by heat denaturation (see Braun *et al*., 2003; Kwa *et al*., 2004) within the native membrane are very similar (see Fig. S2). The similarity, however, no longer holds for the isolated complexes as shown for solubilized neurosporene containing LH2 wt and αWT/βWT_−20A_ (see Fig. S2). The exchange of sphaeroidenone with neurosporene and the replacement of glutamate −20 with alanine both result individually in destabilization of LH2 complex. If the two alterations are combined, the effect is much larger than the sum of the individual effects, indicating that the βGlu-20 and Car's polar moiety synergistically contribute to LH2 stability. However, this becomes obvious only in detergent, when most of the lipids are removed from the complex. Obviously, the surrounding lipids notably contribute in the mutant LH2 to the stability of the LH2 structure. It has not been possible to obtain sufficient amounts for the ESI-MS/MS analysis of isolated LH2 αWT/βWT_−20A_ from DG2 strain by detergent extraction from the membrane owing to the mutants' peculiar membrane properties. A possible role of the Car in LH2–lipid interactions has been suggested previously (Olivera and Niederman, 1993). Perhaps in LH2 wt as opposed to αWT/βWT_−20A_, the surrounding lipids are bound by Glu-20 and the polar moiety of the sphaeroidenone, and therefore not removed by the relatively mild βOG treatment. Alternatively, the alterations result in conformational changes which are less stable, in particular, upon delipidation.

### Alteration of the residue β-glutamate-20 and the Car of LH2 results in altered ICM morphology

**Fig. 6:**
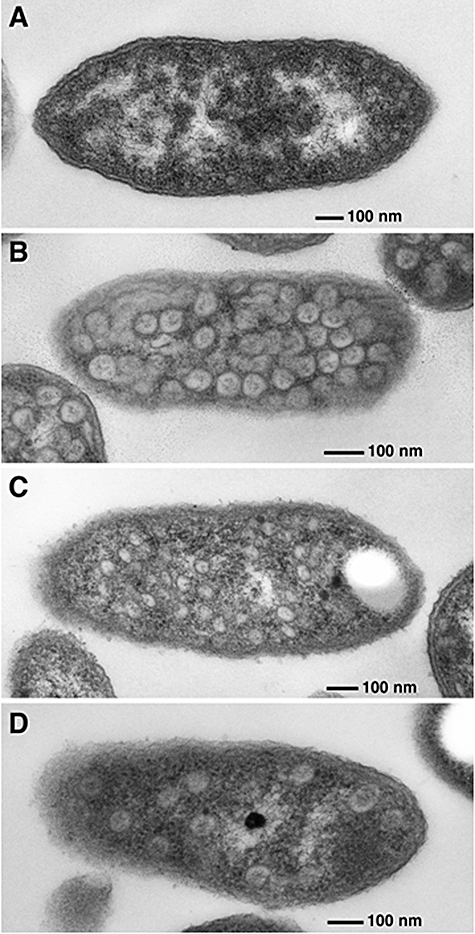
Transmission electron micrograph of ultrathin sections of *R. sphaeroides* strains: DG2 containing LH2 WT (A), carotenoid-less R26.1 (B), DD13 αWT/βWT_−20A_ (C) and DG2 αWT/βWT_−20A_ (D).

To further explore the relation between membrane morphology and LH2, the membranes of LH2 wt and αWT/βWT_−20A_ are compared in *R. sphaeroides* DD13 and DG2 strains. Independent of the major Car, the vesicular ICM containing LH2 wt are of oval shape with sizes ranging from 35 to 55 nm (Figs 2B and 6A). The ICM containing αWT/βWT_−20A_ have also a very similar morphology, indicating that the changes at the LH2–protein–lipid interface upon mutation of glutamate −20 are not resulting in altered membrane morphology in sphaeroidenone-producing cells. On the contrary, the ICM of LH2 αWT/βWT_−20A_ in the neurosporene-producing DG2 strain has a clearly distinct morphology. There are membrane invaginations of the normal oval shape, but there are also substantial numbers of ‘abnormal’ cytoplasmic invaginations and of enlarged vesicular structures ranging from 35 to 90 nm in diameter (Fig. 6D). Thus, the Glu-20Ala mutation and change in Car also affect synergistically the membrane structure. In a previous work it has been shown that decreasing the LH2 content results in enlargement of the chromatophores isolated from *R. sphaeroides* (Sturgis and Niederman, 1996). Duplication of the number of LH2 complexes in these membrane results in a decrease of few nanometers of the average chromatophore diameter (from 37 to 41 nm). The impaired stability of DG2 αWT/βWT_−20A_ (Fig. 6) may result in a reduction of assembled LH2 in the membrane. In order to determine the expression levels of LH2 αWT/βWT_−20A_ both in DD13 and DG2 strains, the content of LH2 complex in the membranes has been compared with the respective total protein content (Table 1). In the membranes of LH2 αWT/βWT_−20A_ containing neurosporene, the total protein content relative to LH2 content is slightly increased (1.5×), indicating a minor reduction in the LH2 expression level as compared with LH2 wt. However, the total protein relative to LH2 is almost as much increased (1.3×) in the membranes of LH2 αWT/βWT_−20A_ containing sphaeroidenone (Fig. 6C). The significant enlargement in diamenter (by ∼40 nm), observed for the vesicular invaginations of DG2 αWT/βWT_−20A_, is thus apparently not due to the slight reduction in LH2 level in the membrane. Interestingly, the morphology of enlarged vesicular ICM invaginations is also observed in the Car-less *R. sphaeroides* R26.1 strain (Fig. 6B) possibly supporting the membrane shaping effect of the Car. The effect on the membrane may be related to the reduced affinity for PE, which may be even further reduced by the alteration in the Car end group in immediate vicinity of βGlu-20. The data are yet insufficient to determine whether the PE–LH2 interaction is altered merely because of the change in carotenoids' chemical structure and aa side-chain or by a conformational change of the N-terminal domain of the LH2. Taken together, these findings suggest that the N-terminal edge of the LH2 complex, comprising glutamate −20 of the β-subunit and the carotenoids' polar head group synergistically contribute to the morphogenesis of the vesicular intracytoplasmatic membranes by specific interactions with adjacent lipids.

What are the molecular features of the LH2 complex that determine the vesicular membrane curvature? Monounsaturated PE molecules are postulated to favour non-bilayer curvature owing to their physico-chemical properties and relative geometries. The postulated PE binding site of the LH2 could function to concentrate PE selectively in the cytoplasmic leaflet. However, induction of positive curvature by only lipid distribution effects would require PE accumulation at the periplasmic bilayer leaflet because of its small head group volume in relation to the acyl tail volume (Chernomordik and Zimmerberg, 1995; Farsad and De Camilli, 2003). In addition, mere lipid distribution effects appear to induce buds of much larger sizes than the typical invagination of the ICMs in *R. sphaeroides* (Sarasij *et al*., 2007).

According to the ‘bilayer-couple’ hypothesis, protein-mediated membrane deformation could be driven by insertion of protein into one leaflet only (Farsad and De Camilli, 2003; Antonny *et al*., 2005). It has been proposed that membrane insertion by an amphipathic helix into one leaflet is sufficient *per se* for facilitating budding events on lipid monolayers (Ford *et al*., 2002). Extending the ‘bilayer-couple’ hypothesis, a possible notion for the lipid dependent membrane shaping effect of the LH2 complex may be envisioned as follows: binding of PE selectively to the N-terminal edge of LH2 should result in the formation of a stably bound lipid rim at the cytoplasmic edge of LH2. Our data suggest that about six PE molecules bind to the isolated LH2 complex. This may even underestimate the actual number of PE bound as some may be removed by the detergent treatment. It has been estimated that 50–100 lipid molecules per LH2 are found in the membrane (Bustamante and Loach, 1994). The PE rim at the N-terminal edge should thus effectively enlarge the surface area of the LH2 complex in the cytoplasmic bilayer leaflet. Thus, the selective binding of PE should induce the bilayer surface imbalance as required for membrane deformation, specifically, the membrane curvature for biogenesis and maintenance of the ICM invagination.

An alternative model for the mechanism of membrane deformation has recently been introduced based on the induction of chirality and tilt of specific lipid molecules in membrane domains (Sarasij *et al*., 2007). Clustering of proteins and lipids into distinct domains has been discussed as a requirement for many cellular budding events. Distinct membrane domains, so-called CM sites, have been proposed to be the site of the assembly of photosynthetic units (Reilly and Niederman, 1985; Koblizek *et al*., 2005). During the stage of LH2 accumulation, CM invagination is stimulated at these sites (Koblizek *et al*., 2005). Accumulation of PE at distinct membrane sites would result in loosely packed lipid head group areas which may promote recognition and partitioning of LH2 (Antonny *et al*., 2005; Mesmin *et al*., 2007). The distinct acyl chain composition of the LH2 associated PE lipids, i.e. more saturated acyl side-chains, also points at distinct lipid domains in which LH2 is enriched. Longer and saturated acyl tails in combination with cholesterol have previously been found to accumulate in membrane microdomains. In prokaryotes, which do not synthesize sterols, however, cholesterol or related compounds are absent. Because of somewhat similar chemical properties, carotenoids may replace cholesterol in their role of formation of lipid domains and preferentially interact with PE molecules that have saturated fatty acyl chains (Wisniewska *et al*., 2003). Interestingly, carotenoids have recently been found, just as cholesterol, in association with long-chain saturated lipids (Wisniewska *et al*., 2003). Accumulation of PE in domains may thus be achieved by specific interactions with Car molecules.

In turn, the specific interactions between the LH2 and the boundary PE lipids may induce ordered arrays of PE with distinct chain tilt or head group orientation at the CM sites. Such ordering has been proposed to drive membrane invaginations of particular size and shape (Sarasij *et al*., 2007). It could effectively be disturbed by replacement of some of the PE by PC molecules as shown to be the case in the mutant LH2 in which glutamate −20 is replaced by alanine. Light inducible chiral macromolecular organization of higher plant LHC-lipid assemblies have previously been reported on in the membrane system of chloroplasts (Garab *et al*., 2000; Simidjiev *et al*., 2004). This long range order of photosynthetic proteins is dependent on specific non-bilayer lipids. The thylakoid membrane is characterized by a high structural stability required for optimal function and at the same time structural flexibility allowing for rapid diffusion of mobile components. This apparent duality has been hypothesized to depend on an optimum protein/lipid ratio which may be effectively regulated by incorporation of high amounts of non-bilayer lipid (Garab *et al*., 2000).

In conclusion, the data presented in this work show that massive mutagenesis of LH2 complex results in changes of the morphology and lipid composition of the intracytoplasmatic membrane of *R. sphaeroides*. These findings extend previous studies which have shown that in LH2 deletion mutants (LH2^-^ LH1^+^ RC^+^) ICM shape is significantly altered; and that onset of photosynthetic growth is accompanied by changes in membrane lipid composition. However, we also discovered that not only the absence of LH2 but changing its protein sequence results in altered membrane properties. The membranes containing such mutants are of tubular shape and contain elevated levels of PC complemented by reduced levels of PE. We revealed that PE is selectively enriched at the LH2–lipid interface of *R. sphaeroides*. Conserved residue, β-glutamate-20, which is located at the N-terminal edge of the TMH, and the adjacent keto carbonyl groups of the complexes' carotenoids make up a potential PE binding site in LH2. Modification of the glutamate and the Car at this site significantly impairs the selective binding of PE. However, the most striking result from this study is that the modifications of the LH2 PE binding site also result in significant changes of the vesicular membrane shape leading to the conclusion that selective binding and crowding of PE at the LH2 lipid interface is essential for maintaining the exact morphology of the surrounding lipid membrane.

## Experimental procedures

### Bacterial strains, plasmids, gene transfer and growth conditions

The bacterial strains used in this work include *Escherichia coli* strain S17-1 [(*thi pro hsdR_ hsdM_ recA* RP4-2 (Tc::mu Kan::Tn7)] and *R. sphaeroides* strain DD13 and DG2 (genomic deletion of both *pucBA* and *pufBALMX*; insertion of SmR and KanR genes respectively) (Jones *et al*., 1992). The mobilizable plasmids used were based on pRKCBC1 (TcR, derivative of pRK415; insertion of a 4.4 kb fragment encompassing *pucBAC*); briefly, this expression vector contains the *pucBA* genes as a 420 bp KpnI–BamHI insert (Jones *et al*., 1992). Growth conditions for *E. coli* and *R. sphaeroides* were as described in (Fowler *et al*., 1995). For *E. coli*, tetracycline was used at concentrations of 10 mg ml^−1^. For *R. sphaeroides*, the antibiotics were tetracycline (1 mg ml^−1^) and neomycin (10 or 20 mg ml^−1^). Conjugative transfer of plasmid from *E. coli* S17-1 to *R. sphaeroides* was performed as described (Fowler *et al*., 1995).

### Construction of mutant LH2

The construction of LH2 αAL_16 4S_/βAL_12_ has been carried out as described previously (Kwa *et al*., 2004). LH2 αWT/βWT_−20Q_, αWT/βWT_−20A_ and αWT/βWT_−20K_ was constructed by site-directed mutagenesis (QuikChange II, Stratagene) by directly mutating *puc*B in pRKCBC1 as described in Garcia-Martin *et al*. (2006).

### Preparation of ICMs

*Rhodobacter sphaeroides* membranes were prepared from cells grown semi-aerobically in the dark by disruption in a French pressure cell and subsequent centrifugation on a sucrose step gradient (Braun *et al*., 2002).

### Thermal denaturation of LH2 membranes

Denaturation was carried out as described in Kwa *et al*. (2004). Purified LH2 membranes were adjusted to A850 = ∼4 cm^−1^ in TE buffer (10 mM Tris, 1 mM EDTA, pH 8.0) and measured in thermostated quartz cuvette. Circular dichroism spectra were recorded with 1 nm s^−1^ scan rate under temperature control. During temperature experiments, the heating rate is 2°C min^−1^, recorded from 15°C to 95°C with integration time of 0.2 s at 845 nm. Data acquisition is done by spectra manager software, analysed, plotted or smoothed by data analysis software Origin 7.0 (OriginLab Cooperation, Northampton, MA, USA).

### Protein quantification

Protein concentrations were determined either by protein assay kits from Fluka advance (Seelze, Germany), Roche ESL (Basel, Switzerland) and Pierce BCATM (Rockford, USA) or from the absorption at 280 nm (absorption coefficient at 280 nm calculated from the amino acid composition, 
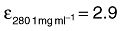
. All the samples were prepared according to the manufacturer's protocol; and repeated more than three times each. For the estimation of the LH2 protein content, the extinction coefficient of B850 BChl was taken as 120 mM^−1^ cm^−1^ (Clayton and Clayton, 1981).

### Modelling of the putative PE binding site

Modelling of the interaction between the β-TMH and Car was carried out by use of the high-resolution data of *Rps. acidophila* (McDermott *et al*., 1995). Replacements of the Car atoms (*Rps. acidophila* > *R. sphaeroides*) and subsequent energy minimizations were done using WebLab Viewer 3.7.

### Electron microscopy analysis

Cells were fixed immediately after collection with 2.5% (v/v) glutardialdehyde (Fisher Scientific Co., Fair Lawn, NJ, USA) in 75 mM sodium cacodylate, 2 mM MgCl2, pH 7.0, for 1 h at room temperature; rinsed several times in fixative buffer and post-fixed for 1 h with 1% osmium tetroxide in fixative buffer at room temperature. After two washing steps in distilled water, the cells were stained en bloc with 1% uranyl acetate in 20% acetone for 30 min Dehydration was performed with a graded acetone series. Samples were then infiltrated and embedded in Spurr's low-viscosity resin (Spurr, 1969). After polymerization, ultra thin sections with thickness between 50 and 70 nm were cut with a diamond knife and mounted on uncoated copper grids. The sections were post-stained with aqueous lead citrate (100 mM, pH 13.0). All micrographs were taken with an EM 912 electron microscope (Zeiss, Oberkochen, Germany) equipped with an integrated OMEGA energy filter operated in the zero loss modes.

### Mass spectrometry

For mass spectrometry analyses, *R. sphaeroides* are grown semiaerobically in the dark at 28°C, and cultures were harvested in their mid-logarithm phase when the absorbance at 650 nm reached 1.2–1.5. To extract total phosholipids, 0.5 ml of sample + 1 ml of methanol was vortexed throughout and left on ice for at least 5 min. Three millilitres of chloroform and 3 ml of water were added and vortexed. Mixtures were centrifuged (3000 *g* for 15 min at 4°C) and the bottom phase was collected and transferred to a clean tube and dried under stream of nitrogen. Lipid profiling of *R. sphaerodies* LH2 samples was carried out by electrospray ionization mass spectroscopy (ESI-MS/MS) essentially as described in Brugger *et al*. (1997). Small sample aliquots (1–10 μl) of cells, chromatophores or isolated LH2 were added to 110 μl of a ammonium acetate (5 mM) in methanol spiked with a mixture of lipid standards (PE/PC/PG). Without prior mixing, the samples were sonicated for 5 min at RT. After mixing precipitated proteins were pelleted at 16000 *g* in a table top centrifuge at 4°C. The supernatant was transferred to another microtube and subjected to mass spectroscopic analysis. Microflow-ESI-MS/MS analysis was performed on a Micromass QII triple-stage quadrupole tandem mass spectrometer equipped with a microflow-ESI source (Z spray) from Micromass (Manchester, UK). Argon was used as collision gas at a nominal pressure of 2.5 × 10^−3^ millibar. The cone voltage was set to 50 V for PC, 45 V for PG and to a cone ramp of 40–65 V in a mass range of mass/charge (m/z) 600–1000 for PE respectively. Resolution of Q1 and Q3 was set to achieve isotope resolution. Quantification of PE was performed by neutral loss scanning, selecting for a neutral loss of 141 (positive ion mode) at a collision energy of 27 eV (1 eV = 1.602 × 10–19 J). PC quantification was performed by precursor ion scanning for fragment ion m/z 184 (positive ion mode, collision energy of 32 eV). PG quantification was performed by precursor ion scanning for fragment ion m/z 171 (negative ion mode) with a collision energy of 37 eV. Unsaturated PE and PG standards were synthesized and purified via HPLC as described (Koivusalo *et al*., 2001). Quantitative analyses were performed as described (Brugger *et al*., 2000; 2004). Phosphate determination was performed according to (Rouser *et al*., 1970). The significance of data was tested by analysis of variance with repeated measures.

### Spectroscopy

UV-visible absorbance spectra were recorded on a Lamda 25 spectrophotometer (PerkinElmer Life Sciences) or Shimadzu UV-2401PC. Circular dichroism measurements were performed on a Dichrograph CD6 (Jobin Yvon, Division Instruments, USA) in 1 mm or 1 cm cylindrical Quartz cuvette or Jasco J715 spectropolorimeter in 1 mm rectangular Quartz cuvette (Hellma, Mühlheim, Germany). The fluorescence excitation spectra of *R. sphaeroides* chromatophores were recorded by Spex FluoroLOG spectrofluorometer (NJ, USA). Excitations were scanned from 300 to 850 nm with the emission wavelength of 880 nm.

## Footnotes

1 A second set of LH2 genes has recently been identified in the genome of *R. sphaeroides* (Zeng *et al.*, 2003). The α-subunits of the second *pucA* gene are absent from assembled LH2 whereas the β-subunits of the second *pucB* are present in LH2 as obvious from MS analysis of purified LH2 complexes (manuscript in preparation, Kwa *et al.*). There appears to be a clear dominance of plasmid-borne β-subunits in LH2 assembly as shown previously (Kwa *et al.,* 2004). The effect of a particular modification is thus clearly reflected in the LH2 properties.

2 The number following BChl indicates the maximum absorption of the red-most absorption band of BChl. The numbering specifies the amino acid position relative to the histidine, designated His 0, which binds the central magnesium of the BChl-B850.

## Supplementary Material

This material is available as part of the online article from:

http://www.blackwell-synergy.com/doi/abs/10.1111/j.1365-2958.2007.06017.x

(This link will take you to the article abstract).
