# Supplementary material for: Mutation of a single residue, β-glutamate-20, alters protein–lipid interactions of light harvesting complex II
Source: Mol Microbiol. 2007 Nov 22;67(1):63–77. doi: 10.1111/j.1365-2958.2007.06017.x (PMC2229836; doi:10.1111/j.1365-2958.2007.06017.x)
Supplement: Supplementary file 1 [file mmi0067-0063-SD1.pdf]

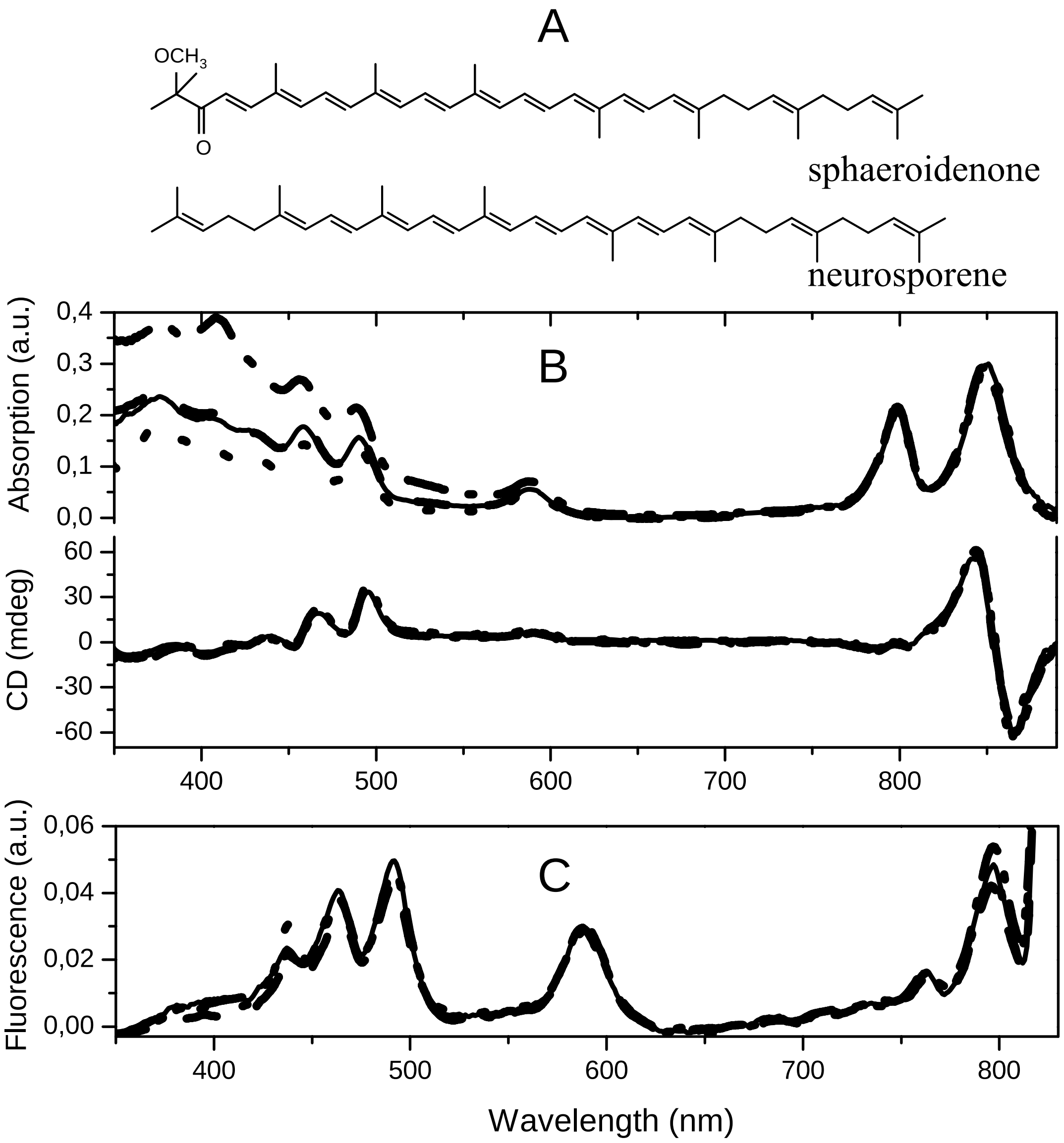

Fig. S1 Functional assembly of LH2 containing neurosporene: WT (—), αWT/βWT-20A (- ● -); or sphaeroidenone: LH2 WT (---), αWT/βWT-20A (···). Spectra are taken of purified membranes. (A) Structures of the carotenoids sphaeroidene (top) and neurosporene (bottom) (B) Absorption spectra (upper trace) and circular dichroism spectra (lower trace). Absorption spectra are normalised at 850 nm. The CD spectra are normalised at the signal maximum at ~843 nm. (C) Fluorescence excitation spectra. Spectra are normalised at ~590 nm.

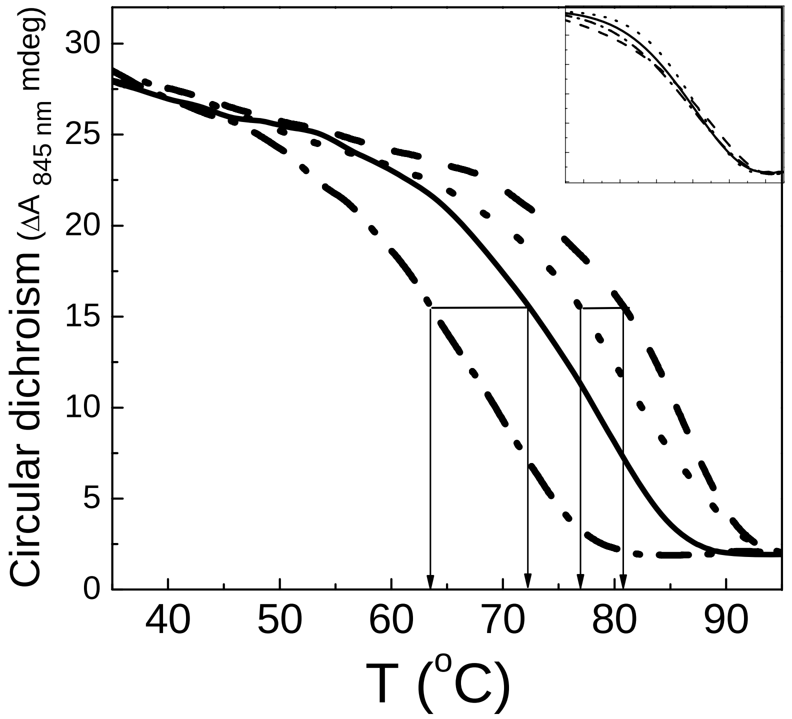

Fig. S2 Thermal denaturation of purified LH2 complexes containing neurosporene: WT (—), αWT/βWT-20A (- ● -); or sphaeroidenone: LH2 WT (---) and αWT/βWT-20A (···). Changes of the CD signal at 845 nm during heating of suspended LH2 membranes. The Tm values are indicated by arrows. The inset depicts the denaturation of LH2 WT (—), αWT/βWT-20A (- ● -) containing neurosporene and LH2 WT (---) and αWT/βWT-20A (···) containing sphaeroidenone in the native membrane.
